# Gallium(III) Complex with Cloxyquin Ligands Induces Ferroptosis in Cancer Cells and Is a Potent Agent against Both Differentiated and Tumorigenic Cancer Stem Rhabdomyosarcoma Cells

**DOI:** 10.1155/2022/3095749

**Published:** 2022-04-23

**Authors:** Monika Hreusova, Vojtech Novohradsky, Lenka Markova, Hana Kostrhunova, Ivan Potočňák, Viktor Brabec, Jana Kasparkova

**Affiliations:** ^1^Czech Academy of Sciences, Institute of Biophysics, Brno-61265, Czech Republic; ^2^Department of Inorganic Chemistry, Institute of Chemistry, P.J. Šafárik University in Košice, Košice 04001, Slovakia; ^3^Department of Biophysics, Faculty of Science, Palacky University in Olomouc, Olomouc 78371, Czech Republic

## Abstract

In this work, gallium(III) complex with cloxyquin (5-chloro-8-quinolinol, HClQ) ligands is shown to effectively inhibit proliferation of rhabdomyosarcoma cells, the frequent, aggressive, and poorly treatable cancer of children. It offers striking selectivity to cancer cells compared to noncancerous human fibroblasts. The data reveal that the complex induces ferroptosis in rhabdomyosarcoma cells, likely due to interfering with iron metabolism. Importantly, it can kill both bulk and stem rhabdomyosarcoma cells. To the best of our knowledge, this is the first compound based on metal other than Fe capable of inducing ferroptosis in cancer cells.

## 1. Introduction

Due to the clinical success of cisplatin and its direct derivatives, metal complexes attract increasing attention as prospective anticancer drugs. Gallium is regarded as the second metal after platinum used for cancer treatment [1]. Gallium nitrate was the first Ga(III) compound approved by the FDA to treat malignancy-associated hypercalcemia. However, its unfavorable properties, such as low therapeutic index and renal toxicity, have prevented its general use in cancer chemotherapy [1–3]. Clinical studies with orally administered gallium(III) chloride showed partial success in treating ovarian cancer. However, no response was observed in patients with lung cancer, likely due to the poor bioavailability of Ga^3+^ [1, 4]. Nevertheless, gallium salts are taken up more selectively by tumors and even more so by metastases if administered orally [1, 4]. Thus, the preclinical and clinical studies revealed the evident potential of Ga(III) anticancer activity, although some disadvantages of simple Ga(III) salts are also apparent.

To circumvent these problems, new Ga(III) complexes consisting of more complex, organic ligands, such as 8-quinolinolate (KP46) [5], maltolate [6], thiosemicarbazones [7], or salen [8], have been prepared and tested. These complexes display greater antitumor efficacy, a broader spectrum of activity, and better bioavailability. Some of these compounds, KP46 and gallium(III) maltolate, have reached clinical trials as anticancer agents, and encouraging results of these trials have been reported [9]. Recently, Ga(III) complexes containing polypyridyl ligands were also shown to kill both bulk and cancer stem osteosarcoma cells effectively. In addition, these compounds have been shown to induce apoptosis in osteosarcoma cells via damaging genomic DNA [10]. Thus, coordination of gallium with organic ligands proves to be a promising strategy for designing therapeutic agents with several advantages over the metal-based drugs currently used in the clinic.

For enhanced treatment efficacy and limitation of acquired resistance, the current trend in clinical research has shifted from monotherapy to combination therapy [11]. For instance, a major objective of discovering KP46 was its application for combination therapy with cisplatin [12], and this combination was proved to act synergistically in ovarian and colon carcinomas [13] as well as in osteosarcoma [14]. In addition, an oral formulation of KP46 showed synergism with a broad range of antitumor agents targeting the endoplasmic reticulum in multiple tumor types [15]. However, when combining drugs, a problem arises that different drugs have different solubility and pharmacokinetics [16, 17]. Therefore, the design of new single agents combining in one molecule two or more moieties with their own bioactive properties can be a promising strategy, more profitable than a combination of two or more individual drugs.

Cloxyquin (5-chloro-8-quinolinol, HClQ) is a well-known anti-infective and antibacterial drug clinically used to treat tuberculosis. It displays antiproliferative activity in human ovarian and lung cells through allosteric proteasome inhibition [18]. HClQ has a unique p53-modulating activity that shifts its transactivation from proapoptotic to protective responses, including enhancing p21 induction, thereby protecting mice from *γ*-irradiation-induced gastrointestinal death [19]. In addition, HClQ protects from cardiac ischemia-reperfusion injury in mice by uncoupling mitochondria and inducing autophagy [20]. HClQ also possesses strong antimelanoma properties *in vitro* and *in vivo* mediated by peroxisome proliferator-activated receptor-gamma (PPAR*γ*) [21].

Therefore, gallium(III) complexes with ClQ and other 8-quinolinolato halo derivatives were recently prepared, characterized by elemental analysis, IR and NMR spectroscopy, X-ray structure analysis, and tested against selected cancer cell lines [22]. The data have revealed that [Ga(ClQ)_3_] exhibits propitious antiproliferative activity with a high level of selectivity toward cancer over noncancerous cells, highlighting this complex as a candidate for further mechanistic studies.

**Figure 1:**
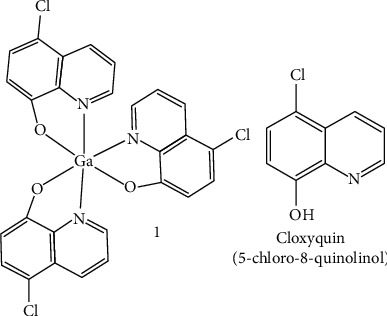
Schematic representation of compounds investigated in this work.

In this work, we report the remarkable antiproliferative activity of this new tris-(5-chloro-8-quinolinolato)gallium(III) complex (**1**, Figure 1), particularly against rhabdomyosarcoma (RD) cells, one of the most problematic and poorly treatable pediatric tumors. Notably, the new Ga-complex shows its activity in both cancer stem cells (CSCs) and bulk, differentiated cancer cells simultaneously. Additionally, our data indicate that the combination of both Ga metal center and cloxyquin ligands presented in the molecule of complex **1** is responsible for this drug's unique mechanism of action, not achievable for the individual components.

## 2. Materials and Methods

### 2.1. The Chemicals

Complex **1** was synthesized and characterized by the method described in our recently published article [22]. The purity was ∼98%. HClQ, cisplatin, carboplatin, and Ga(NO_3_)_3_ were purchased from Sigma (Prague, Czech Republic). MTT (3-(4,5-dimethylthiazol-2-yl)-2,5-diphenyltetrazolium bromide) was from Calbiochem (Darmstadt, Germany).

### 2.2. Cell Cultures

The human rhabdomyosarcoma (RD) cells were purchased from the American Type Culture Collection (ATCC, Manassas, VA, USA), human colorectal carcinoma cells HCT116 and human breast cancer MCF-7 cells were kindly supplied by Professor B. Keppler, University of Vienna (Austria), human melanoma 518A2 were kindly supplied by prof. R. Schobert, University of Bayreuth (Germany). In addition, highly invasive breast carcinoma MDA-MB-231 cells and human MRC5pd30 cells derived from normal lung tissue were purchased from the European Collection of Authenticated Cell Cultures (ECACC) (Salisbury, UK). RD cells were grown in RPMI 1640 medium (Biosera, Boussens, France), the other cells were kept in DMEM medium (high glucose, 4.5 gL^−1^, PAA, Pasching, Austria); both media were supplemented with gentamycin (50 mgmL^−1^, Serva, Heidelberg, Germany) and 10% heat-inactivated fetal bovine serum (PAA, Pasching, Austria). All cell lines were cultured in a humidified atmosphere (5% CO_2_) at 37°C and subcultured two to three times a week.

### 2.3. In Vitro Antiproliferative Assay

The effects on cell proliferation were evaluated by commonly used colorimetric MTT assay. Briefly, the cells were seeded in 96-well tissue culture plates at an appropriate density (5 × 10^3^ cells/well in 100 *μ*L of medium for RD, MDA-MB-231, MCF-7, 518A2, MRC5pd30 and 2 × 10^3^ cells/well in 100 *μ*L of medium for HCT116). After overnight incubation, the cells were treated with the tested compounds in a final volume of 200 *μ*L/well for an additional 72 h. The stock solutions were freshly prepared in DMSO or water and subsequently diluted into the culture medium so that the final concentration of DMSO did not exceed 1%. Concentrations of the Ga or Pt in the medium during the treatment were verified by ICP-MS. After the incubation period, a freshly diluted MTT solution (20 *μ*L, 1.25 mgmL^−1^ in PBS) was added to each well, and the plates were incubated for another 4 h. After removing the medium, the precipitated formazan product was dissolved in 100 *μ*L of DMSO. Experiments were evaluated by measuring the absorbance at 570 nm (reference wavelength was 620 nm) using an absorbance reader Tekan Spark (Switzerland). The values of IC_50_ were calculated from curves constructed by plotting relative absorbance (related to control, untreated cells) versus drug concentration.

### 2.4. Cellular Uptake

Cellular accumulation of gallium from Ga-complex **1** and Ga(NO_3_)_3_ was determined as already described [23]. In brief, 2 × 10^6^ RD cells were seeded on 100 mm Petri dishes and kept in a drug-free medium at 37°C in a 5% CO_2_ humidified atmosphere overnight. Then, the cells were treated with compounds at their 10 *μ*M concentrations in the cell growth medium and allowed drug exposure for 5 h. The treatment under these conditions had no significant impact on the viability of RD cells, as measured by the Trypan blue exclusion test (the viability of cells after the treatment was more than 95% for all samples). The cells were subsequently detached using 0.25% trypsin, washed thoroughly with ice-cold PBS, collected by centrifugation, and counted using an automatic cell counter (BioRad TC10). Finally, the cell pellets were digested, and the quantity of metals taken up by the cells was determined by ICP-MS (Agilent Technologies, CA, USA). External calibration was used; analyses included a set of calibration reference samples [in the range of 0.05–1000 *μ*gL^−1^ Ga (Astasol Mix in 5% HNO_3_ v/v from Analytica, Czech Republic)] to generate a calibration curve of instrument response which could be correlated to actual gallium concentration within the investigated samples.

### 2.5. Subcellular Localization of Complex 1

RD cells were seeded on the confocal 35 mm glass-bottom dishes (Mattek, Ashland, USA) at the density of 1.5 × 10^5^ Cells/dish. Cells were cultured overnight and then treated with **1** (10 *μ*M). After the treatment (3 or 24 h), the medium was removed, and cells were carefully washed. Subsequently, a drug-free medium was added, and samples were visualized on the SP8 SMD laser scanning confocal microscope [Leica Microsystems, Wetzlar, Germany (*λ*_ex_ 405 nm, detection channel ranging from 500 to 600 nm)]. Control, untreated cell samples were also analyzed to exclude possible auto-fluorescence of the cells.

### 2.6. Cell Death Detection

For propidium iodide/annexin-V staining, the cells were treated with indicated concentrations of **1** or HClQ for 24 h. As for positive controls, the cells were treated with 10% EtOH for 1 h (necrosis) or with 2 *μ*M staurosporine for 4 h (apoptosis). Afterward, the cells were collected, and washed thoroughly in PBS (4°C) and subsequently stained with PI (1 *μ*gmL^−1^) and annexin-V Pacific Blue conjugate (5 *μ*L per 100 *μ*L of the cell suspension, Thermo Fischer Scientific) for 15 min at room temperature. Cells were analyzed immediately after staining by flow cytometry (BD FACSVerse), and data were analyzed using FCS Express 6 software (DeNovo software; Glendale, CA). Dot plots representative of three independent experiments with similar results are shown.

For autophagy detection, the cells were treated with **1** or HClQ at their equitoxic concentrations corresponding to one-, two-, or fourfold IC_50,72h_ for 24 h or kept untreated (negative control). As for the positive control, a mixture of rapamycin (1 *μ*M) +chloroquine (100 *μ*M) was added to the cells and incubated for 24 h. Then, the cells were washed in PBS (4°C), stained with a CYTO-ID® autophagy detection kit (Enzo Life Sciences) for 30 min, and analyzed by flow cytometry (BD FACSVerse). This autophagy detection kit measures autophagic vacuoles and monitors autophagic flux in lysosomally inhibited live cells using a dye that selectively labels accumulated autophagic vacuoles.

### 2.7. Effect of Specific Inhibitors of Cell Death

The effect of specific inhibitors of unregulated necrosis and necroptotic pathways on the cell death induced by **1** was assayed by measuring propidium iodide uptake. RD cells grown in six-well plates (2.5 × 10^5^ cells/well) were treated with **1** (15 *μ*M) in the presence or absence of inhibitors of the same concentrations as already described [24] for 24 h. Afterward, the cells were washed with PBS (3 × 1 mL), harvested, incubated with PI (3.3 *μ*gmL^−1^ for 20 min), and analyzed by a flow cytometer (BD FACSVerse, BD Biosciences, San Jose, USA). Cell populations were analyzed using FCS Express 6 software (DeNovo software; Glendale, CA).

### 2.8. Determination of ROS

RD cells were seeded on the 6-well plates at the density of 2.5 × 10^5^ cells/well and incubated overnight. Then, the cells were treated with increasing concentrations of **1** or HCIQ corresponding to 0, 5, 10, and 20 *μ*M. After 3 h of incubation with the tested compounds, cells were washed three times with PBS. The wells were loaded with the staining solution of CellRox®-deep red (Thermo-Fisher Scientific) according to the manufacturer's protocol. The cells were then washed three times with PBS, harvested to the tubes, and analyzed by the flow cytometer BD FACS Verse. Acquisition of the samples was set to 30,000 events from a single cell population. Data were analyzed using FCS Express 7 (DeNovo software; Glendale, CA).

### 2.9. Western Blotting

RD cells were treated as indicated in the section *In Vitro* Antiproliferative Assay. The cells were scraped, washed, pelleted by centrifugation, and lysed for 1 h with ice-cold RIPA buffer supplemented with PMSF, sodium orthovanadate, and protease inhibitor cocktail according to the manufacturer's protocol (Santa Cruz Biotechnology, INC.) The extracts were cleared (15,000 g; 10 min) and combined with 2 × LBS buffer (4% SDS; 10% 2-mercaptoethanol; 20% glycerol; 0.125 M Tris.HCl and 0.004% bromophenol blue) and heated for 5 min at 95°C. The samples were separated by SDS-PAGE (4–15%; Mini-PROTEAN® TGX^TM^ Precast Gels), transferred to PVDF membrane, and GPX4, Trf1, and GAPDH were detected using specific primary (Anti-Transferrin Receptor antibody (Abcam, ab214039; 1 : 1000), Anti-Glutathione Peroxidase 4 antibody (Abcam, ab125066; 1 : 1000), Anti-GAPDH antibody (Sigma-Aldrich, G8795; 1 : 200)) and secondary antibodies (Goat Anti-Rabbit IgG (HRP) (Abcam, ab205718; 1 : 1000), and Goat Anti-Mouse IgG (HRP) (ThermoFisher Scientific, 32430; 1 : 200). After adding the substrate (SignalFire^TM^ ECL Reagent (A+B)), the luminescence was recorded with the Amersham Imager 680. The densitometric evaluation was performed using Aida image software.

### 2.10. Lipid Peroxidation Assessment

RD cells were seeded on the 6-well plate at the density of 2.5 × 10^5^ cells/well and incubated overnight. Then, the cells were treated with an increasing concentration of the tested compounds for 3 h. Menadione was added as the positive control at the concentration of 100 *μ*M [25]. After the treatment, samples were stained with Bodipy™ 665/676 dye (ThermoFisher Scientific) at the final concentration of 5 *μ*M in PBS and incubated for 30 min in a humidified CO_2_ incubator. Cells were washed three times with PBS, harvested, and subsequently analyzed by flow cytometry (BD FACS Verse). Data were analyzed using FCS Express 7 (DeNovo software; Glendale, CA).

### 2.11. Cell Sorting

Primary culture of RD cells was stained for the surface CSC-markers CD133 antibodies conjugated with APC fluorochrome and subsequently sorted with magnetic anti-APC microbeads, a method based on anti-fluorophore microbeads from Miltenyi Biotec (Gladbach, Germany) as already described [26]. Briefly, RD cells were stained with CD133-APC for 15 min at 4°C, then incubated with anti-APC microbeads for 15 min at 4°C and magnetically sorted using MS columns. RD CD133+ were maintained as 3D cultures in ultra-low attachment conditions to suppress their differentiation. The CSC-depleted subpopulations of cells not expressing CD133 markers (RD CD133-) were also sorted and used for comparative purposes. Three-dimensional (3D) cultures were maintained on ultra-low adherent S7 plastics from Corning (NY, USA) and cultured in DMEM-F12 ham medium supplemented with B27 (2%; Thermo Fisher Scientific, MA, USA), 0.15% BSA, EGF (Sigma; 10 ngmL^−1^), and FGF-2 (Sigma; 20 ngmL^−1^).

### 2.12. Antiproliferative Activity in 3D Spheroids

Single cells gained from magnetic sorting were placed to ultra-low attachment conditions and DMEM-F12 ham medium supplemented with B27 (2%; Thermo Fisher Scientific, MA, USA), 0.15% BSA, EGF (Sigma; 10 ngmL^−1^), and FGF-2 (Sigma; 20 ngmL^−1^), cultured for 96 h to form spheroids and then treated with tested compounds for further 72 h. After the incubation, samples were assayed with CellTiter-Glo® 3D (Promega, WI, USA), and the luminescence signal was detected on Infinite M200 (Tecan, Manedorf, Switzerland). The IC_50_ values were determined from the curves constructed by plotting the reading luminescence signals versus drug concentrations. Samples were photographed by Olympus CKX41 inverted microscope with 10X/0.25 phase contrast objective. Digital images were acquired and analyzed by the QuickPHOTO MICRO 3.1 program (PROMICRA, Prague, Czech Republic).

## 3. Results and Discussion

### 3.1. Antiproliferative Activity

Antiproliferative activity of complex **1** was studied by commonly used MTT assay after 72 h of incubation with a set of human cancer cell lines of various origins—breast, colon, melanoma, and mesenchymal cells (see Table 1). Moreover, the human noncancerous lung fibroblasts were also included in the study. For comparative purposes, the identical tests were also performed with clinically approved platinum(II) anticancer drugs, cisplatin, and carboplatin.

The data revealed that complex **1** showed potency in the micromolar scale (Table 1). Notably, **1** displayed 1.5- to 92-fold greater potency in MDA-MB-231, HCT116, 518A2, and RD cancer cells than clinically approved cisplatin or carboplatin. On the other hand, it was less potent than cisplatin or carboplatin in MCF-7 cancer cells. In addition, the experiments also confirm very low activity in noncancerous skin fibroblasts, thus indicating superior selectivity for cancer over noncancerous human cells.

Control studies also showed that the potency of Ga(NO_3_)_3_ and free ligand cloxyquin toward cancer cells was markedly lower than that of **1** (Table 1). This suggests that the intact **1** is likely to be responsible for the observed antiproliferative activity rather than its components. Notably, a markedly higher IC_50_ value (IC_50_ is defined as a compound concentration that induces 50% cell growth inhibition) obtained for **1** was observed in MCF-7 compared to those in other cell lines. As discussed in section on antiproliferative activity of **1** in MCF-7 cells, this observation is likely a consequence of the fact that these cells are less vulnerable to ferroptosis.

A detailed inspection of data in Table 1 revealed the highest activity of **1** in rhabdomyosarcoma (RD) cells, being approximately 2–71 times more effective than in the other cancer cell lines (Table 1). Rhabdomyosarcoma is a highly aggressive cancer of mesenchymal origin. This malignancy is the most frequent soft tissue sarcomas of childhood and adolescence and the third most common solid tumor [27]. It can occur in any soft tissue site in the body but is primarily found in the head, neck, orbit, and genitourinary tract. Unfortunately, despite intensive clinical trials conducted in the last several decades, outcomes for patients with this disease have not significantly improved. The survival of metastatic or relapsed disease remains extremely poor (survival rates less than 20%) [28]. Consequently, developing a potent treatment for this aggressively recurrent disease represents an important task. Therefore, the following studies aimed to understand the effect of **1** in RD cells were performed.

### 3.2. Accumulation in Rhabdomyosarcoma Cells

To shed light on the mechanism of action of the gallium complex **1** in RD cells, intracellular accumulation of **1** was measured to estimate rhabdomyosarcoma cell permeability. RD cells were treated with **1** and Ga(NO_3_)_3_ at a nonlethal dose (10 *μ*M for 5 h), and the internalized gallium content was determined by inductively coupled plasma mass spectrometry (ICP-MS). It was verified by trypan blue assay that the populations of cells after treatment contained no more than 5% of dead cells (95% viability).

As shown in Table 2, 14 ± 1 ng of Ga per million cells were taken up and accumulated in RD cells treated with **1**, which is ca. 16-fold more than in the case of Ga(NO_3_)_3_. Thus, it suggests that the presence of cloxyquin ligands in the molecule of **1** enhances the accumulation of Ga in cells, likely due to the facilitating transport through the phospholipid cell membrane.

The fluorescence properties of **1** (Figure S1) allowed us to monitor cellular distribution in living cells. RD cells were incubated with **1** (10 *μ*M) for 3 and 24 h, carefully washed, and subjected to imaging by fluorescence confocal microscopy. The distribution of fluorescence signal from **1** was analyzed. The emission window on the confocal microscope was set strictly to the fluorescence emission wavelengths of **1**; thus, the detected signal can be attributed only to **1**. Moreover, bright-field images of the cells were taken as well to prove the viable state of cells and determine cellular morphology.

**Figure 2:**
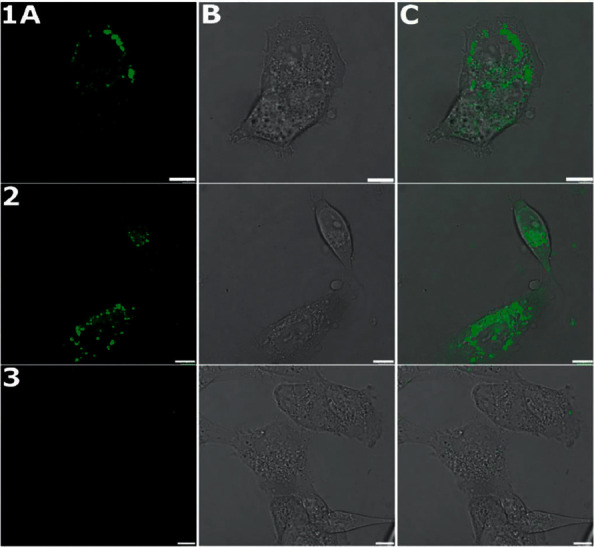
Localization of **1** in RD cells obtained by confocal microscopy. Cells were treated with complex **1** (10 *μ*M) and incubated for 3 h (line 1) or 24 h (line 2). Untreated cells (line 3) were included as a control of autofluorescence of RD cells. Images were obtained in fluorescence mode (column A), bright field mode (column B), or overlaid (column C). Scale bars represent 10 *μ*m.

As indicated in Figure 2, **1** was visualized in living cells and was found predominantly in the cytoplasm. It suggests that nuclear DNA is unlikely a primary molecular target of **1**. Moreover, fluorescence microscopy revealed that **1** was stable for at least 24 h in the intracellular environment since the free ligand exhibited negligible fluorescence intensity (Figure S1) [29].

### 3.3. Mechanism of Rhabdomyosarcoma Cell Death Treated with 1

Detailed subcellular analysis of fluorescent signal also unveiled a localization of **1** in discrete cytoplasmic puncta, presumably peroxisome-like structures or multivesicular bodies [30]. Time-lapse confocal microscopy showed an initial perinuclear puncta assembly 3 h after the treatment (Figure 2, line 1) followed by redistribution of peroxisome-like structures to the whole cytoplasm (Figure 2, line 2). Although the morphology of the cells was affected by incubation with **1** (particularly after 24 h of treatment), the treated cells surprisingly exhibited none of the morphological features typical for cells undergoing apoptosis.

**Figure 3:**
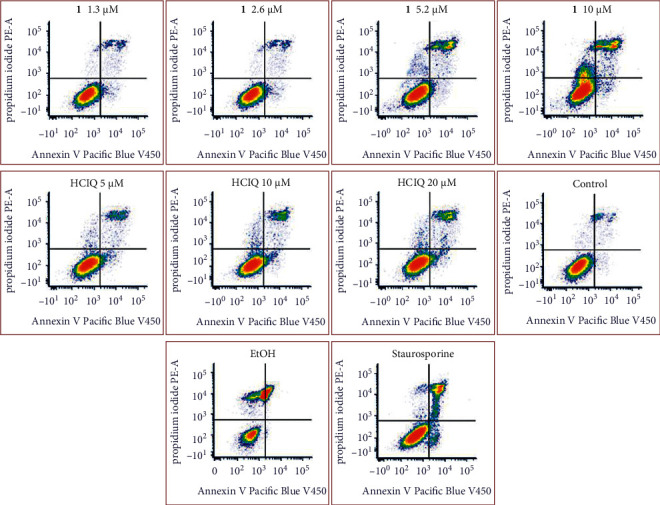
Representative density plots of RD cells after their PI/annexin V Pacific blue staining. The cells were treated for 24 h with **1** or ClQ at their equitoxic concentrations, corresponding to one-, two-, and fourfold IC_50,72h_. Early apoptotic cells are in the right lower quadrant (annexin V-positive, PI-negative), whereas cells undergoing necrotic processes are in the left upper quadrant (annexin V-negative, and PI-positive). The signals in the right upper quadrant (annexin V and PI-positive) represent dead (necrotic and late apoptotic) cells. The well-established specific cell-death inducers staurosporine (apoptosis) and EtOH (nonspecific necrosis) have also been included in the experiment as positive controls.

Therefore, flow cytometry was employed to determine the cellular response to **1** more deeply. Annexin V-propidium iodide (PI) dual staining assay showed (Figure 3) that the treatment of RD cells with **1** for 24 h induced a concentration-dependent increase in both annexin V-negative/PI-positive and annexin V-positive/PI-positive cell population (early necrotic cells and cells in the late period of cell death, respectively). In contrast, the annexin V-positive/PI negative cell population constituting the fraction of early apoptotic cells remained almost unchanged (Figure 3) even at a relatively high concentration of **1**, indicating that **1** does not induce a significant apoptotic response in RD cells. In contrast, free ligand (cloxyquin) slightly increases both early necrotic and early apoptotic populations in a concentration-dependent manner.

The finding that **1** induces in RD cells other than apoptotic response prompted us to determine the mode of the cell death provoked by **1**. Besides apoptosis and unregulated necrosis, autophagy-dependent cell death has been recognized [31]. Moreover, many novel forms of nonapoptotic regulated cell death have been identified, including necroptosis (regulated necrosis), ferroptosis, entotic cell death, netotic cell death, parthanatos, lysosome-dependent cell death, alkaliptosis, and oxeiptosis [31]. Thus, further experiments have been aimed to understand cell death processes provoked by **1** and determine the exact mechanism involved.

**Figure 4:**
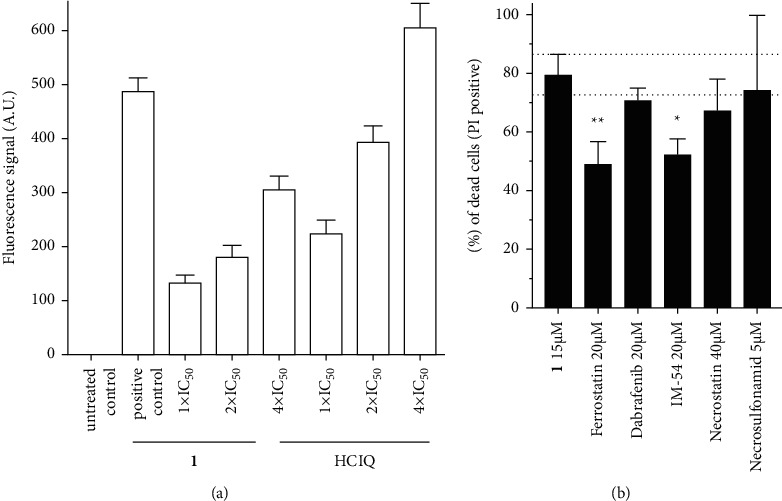
Flow cytometric analysis of the mechanism of cell death after the treatment with **1** in RD cells: (a) Quantitative evaluation of fluorescence signal from Cyto ID Autophagy Detection reagent. Cells were treated with the mixture of 1 *μ*M rapamycin + 100 *μ*M chloroquine (positive control), **1** or HClQ at the concentrations corresponding to one-, two-, or fourfold IC_50_,_72_ _h_ for 24 h. The bars represent the arithmetic mean of fluorescence signal ± SEM, *n* = 3. Fluorescence signal from untreated cells (negative control) was subtracted. (b) Graphical representation of dead cell population (%) when treated with **1** in the absence and presence of ferrostatin (20 *μ*M), dabrafenib (20 *μ*M), IM-54 (20 *μ*M), necrostatin-1 (40 *μ*M), and necrosulfonamid (5 *μ*M), after 72 h coincubation. Error bars represent standard deviations, *n* = 3. The symbols (^*∗*^ and ^*∗∗*^) denote significant differences (*p* < 0.05 and *p* < 0.01, respectively) from the sample incubated with **1** in the absence of the inhibitor.

The levels of autophagy in RD cells were detected with the aid of Cyto-ID® Autophagy Detection Kit (Enzo) using flow cytometry. It was verified that the inherent fluorescence of **1** did not affect the final analysis (Figure S2). As shown in Figures 4(a) and S2, RD cells exhibited a significant induction of autophagy in response to cloxyquin, in agreement with already published data [20, 32]. Similarly, the level of autophagy was also increased in cells treated with **1**, although it was less pronounced than the response to free ligand. Considering a significantly higher overall activity of **1** compared with free cloxyquin, the data suggest that although **1** partially activates an autophagic response in human RD cells, autophagy is not the only pathway leading to cell death induced by **1**. Thus, another mechanism is likely to contribute to the overall activity of **1** in RD cells.

To distinguish between different forms of nonapoptotic cell death, specific inhibitors have been employed, such as ferrostatin (a specific inhibitor of ferroptosis), dabrafenib (selective for Raf kinase essential for necroptosis), IM-54 (a selective inhibitor of necrosis induced by oxidative stress), necrostatin (inhibits TNF-*α*-induced necroptosis), and necrosulfonamid (inhibits MLKL-mediated necroptosis). The cells were coincubated with **1** in the absence or presence of a particular inhibitor, and the fraction of dead (PI-positive) cells in the population was assessed using flow cytometry. It was verified that the treatment with indicated concentrations of the inhibitors alone did not affect the viability of cells. The results showed that coincubation of **1** with inhibitors of various necroptotic pathways (dabrafenib, necrostatin, and necrosulfonamid) did not significantly affect the potency of **1** to kill RD cells (Figure 4(b)), confirming necroptosis as insignificant in the mechanism of action of **1**. In contrast, coincubation with ferrostatin reduced the potency of **1** in RD cells (Figure 4(b)), providing evidence for the ability of complex **1** to kill RD cells by ferroptotic mechanism. Additionally, IM-54, which selectively blocks oxidative stress-induced necrotic cell death, was also effective in inhibiting the antiproliferative activity of **1**. It suggests that oxidative stress also plays an important role in the mechanism of action of **1**. It has been shown that ferroptosis induction is accompanied by processes resulting in a large amount of ROS, which promotes ferroptosis [33]. Thus, the effect of IM-54 observed in this experiment could be attributed to reducing oxidative stress accompanying ferroptosis.

Moreover, the data from the localization study by confocal microscope described above revealed localization of **1** in discrete peroxisome-like structures or multivesicular lysosome-related vacuoles (Figure 2). Recent studies show that peroxisomes play a crucial role in ferroptosis through the biogenesis of plasmalogens for lipid peroxidation [34]. Already published data also indicate that elevated levels of autophagy [35] and accumulation of autophagosomes and other lysosome-related vacuoles [36] are observed in ferroptotic cells. Taken together, all presented results strongly support the view that ferroptosis is a prominent mode of programmed death induced by **1** in RD cells, along with autophagy.

### 3.4. Production of ROS in Rhabdomyosarcoma Cells Treated with 1

**Figure 5:**
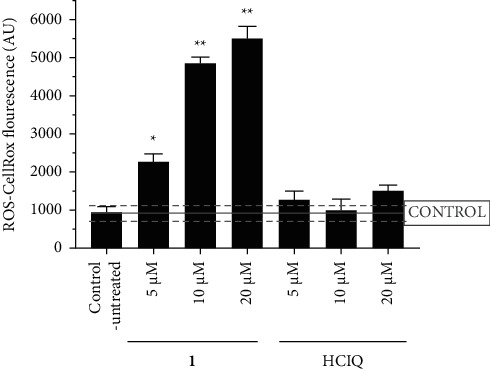
Quantification of ROS generated in RD cells after the treatment with the investigated compounds. The bar chart depicted an analysis of the mean fluorescence signal from the CellRox reagent. Statistical analysis was calculated using unpaired *t*-test, and the statistically significant bars were marked with ^*∗*^*p* ≤ 0.05 or ^*∗∗*^*p* ≤ 0.01.

To further support the hypothesis that ferroptosis is a prominent mode of programmed death induced by **1** in RD cells, the level of ROS in RD cells treated with **1** was measured. The accumulation of reactive oxygen species, leading to lipid and polyunsaturated fatty acids peroxidation, is a typical hallmark of ferroptosis [36]. Cells were treated with **1** for 3 h, and the level of oxidative stress was analyzed by CellRox reagent with subsequent analysis by flow cytometry. Data showed a significant increase in ROS level after the treatment with **1** (Figures 5 and S3(a)). As indicated, stimulation of ROS formation was concentration-dependent.

On the other hand, nonsignificant changes in the level of ROS were observed after the treatment with HCIQ (Figures 5 and S3(b)). These changes were below the significance of implemented statistical analysis throughout all treated concentrations. Overall, the data indicate a relatively high production of ROS after the cells were treated with **1**, suggesting their role in initiating lipid peroxidation and stimulating ferroptosis. Since HClQ is ineffective in this respect, the elevation of ROS concentration can be attributed to the presence of metal (Ga(III)) in the molecule of **1**.

**Figure 6:**
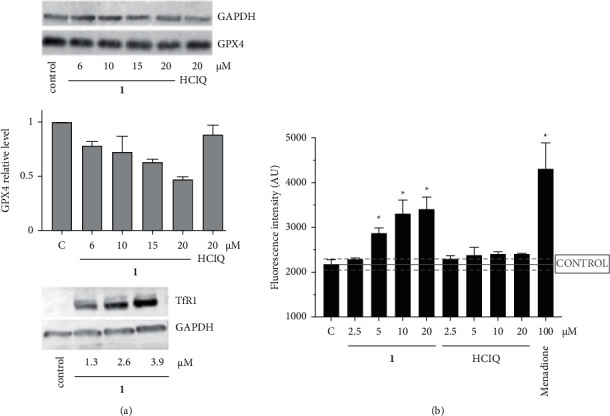
(a). Western-blot analysis of GPX4 and TfR1 levels. Representative membranes are shown. Middle panel: (a) Quantitative analysis of GPX4 level (relative to loading control GAPDH); the level of GPX4 in untreated cells is taken as 1. Bars represent a mean ± SEM of two independent experiments. (b) Quantification of lipid peroxidation in RD cells determined by flow cytometry. Data were subjected to statistical analysis using nonparametric student's *t*-test, and the significant data were marked with ( ^*∗*^ = *p* ≤ 0.05).

To further support the role of ferroptosis in cell death induced by **1**, the other features generally accepted as hallmarks of this type of cells death were analyzed. Glutathione peroxidase 4 (GPX4) plays a pivotal role in ferroptosis and is the key regulator of its occurrence, mainly by inhibiting the formation of lipid peroxides. Inhibition of GPX4 activity can lead to the accumulation of lipid peroxides, which is a marker of ferroptosis [37]. Thus, the inhibition of GPX4 (via inactivation or degradation) accompanied by an accumulation of lipid peroxides is the critical step in ferroptosis [38]. Western blotting analysis (Figure 6(a)) revealed that the GPX4 level was reduced in RD cells treated with **1** in a concentration-dependent manner. However, GPX 4 level in cells treated with HClQ alone was affected insignificantly. Moreover, treatment with **1** stimulated expression of the transferrin-1 receptor (TfR1, Figure 6(a)), which is also a feature typical for the ferroptotic pathway [39].

Lipid peroxidation in cells treated with **1** was measured by sensor dye Bodipy™ 665/676 because the mechanism of cell death by ferroptosis is characterized and driven by the accumulation of lethal lipid peroxides and disturbed lipid metabolism [40]. Notably, the highly conjugated polyene system of Bodipy™665/676 possesses suitable fluorescent characteristics [41, 42], eliminating possible overlaps between the fluorescent signals of probe and **1**. The resulting data indicate a significant fluorescence increase after treating RD cells with **1** (Figures 6(b) and S4(a)). However, the treatment of the cells with HClQ does not affect the intensity of fluorescence signal that remained at the level of untreated control for all the concentrations of HClQ (Figures 6(b) and S4(b)). Thus, the analysis confirmed that **1**, but not free HClQ, potentiates the accumulation of highly toxic lipid peroxides in cells, which have an important impact in triggering and initiation of ferroptosis.

### 3.5. Antiproliferative Activity of 1 in MCF-7 Cells

The important role of ferroptosis in the cellular response to **1** can be further supported by its relatively low activity in MCF-7 Cells (Table 1). These cells overexpress GPX4, making them less vulnerable to ferroptosis [43]. Thus, the markedly higher IC_50_ value in MCF-7 compared to those in other cell lines (Table 1) may be a consequence of this factor.

### 3.6. Antiproliferative Activity of 1 in Rhabdomyosarcoma Cancer Stem Cells

Notably, childhood rhabdomyosarcoma is derived from muscle cells that failed to differentiate fully, emphasizing the important role of cancer stem cells (CSCs) in this type of malignancy.

Therefore, further studies were intended to verify whether **1** shows activity in stem-like RD cells.

To isolate CSCs from different tumors and tumor cell lines, expression of cell surface markers, epithelial-specific antigens are commonly exploited. A transmembrane protein CD133, also known as Prominin1, has been suggested as a CSC marker in rhabdomyosarcoma [28, 44]. Therefore, a fraction of RD cells expressing CD133 (RD CD133+) was sorted out as indicated in the experimental section and tested with regard to the ability of **1** to inhibit the growth of these CSCs-like RD cells. The sensitivity of these cells was compared with that of differentiated, CD133 nonexpressing fraction of RD cells (RD CD133-).

The sorted cells (both CD133 positive and negative) were cultured for 96 h to form spheroids (Rhabdospheres) and then treated with **1** for another 72 h. The viability of cells was determined by using Cell TiterGlo 3D assay. The 3D culture can render more predictive results than conventional 2D cell cultures, as it better reflects the tumor microenvironment, including nutrient and oxygen gradients, drug penetration, and intercellular interactions. Cyclophosphamide, a clinically used agent to treat most childhood solid tumors, including rhabdomyosarcoma (also in combination with other chemotherapeutics) [45, 46], was used for comparative purposes.

The results (Table 3) indicated that **1** showed in rhabdospheres the IC_50_ values in micromolar concentrations. Notably, both fractions of RD tumorspheres were similarly sensitive to the treatment with **1**. In contrast, clinically used cyclophosphamide showed under the same experimental conditions relatively high IC_50_ values, being five- to ninefold less potent than complex **1**.

The effects of **1**, HClQ, and Ga(NO_3_)_3_ on the morphology of rhabdospheres formed from the suspension of RD CD133+ and RD CD133– single cells are shown in Figure S5. Control, untreated rhabdospheres have round-shaped morphology with a well-defined surrounding edge (Figure S5, left top panels). In contrast, the treatment with **1** considerably reduced the size of spheroids, indicating the ability of **1** to inhibit rhabdosphere growth. In addition, the spheroids treated with **1** displayed heterogeneous morphology with dissociated cell clumps (Figure S5, right top panels). The effect of free HClQ was less pronounced (Figure 6, left bottom panels). In contrast, gallium(III) nitrate treatment has no significant impact even at its very high (500 *μ*M) concentration. The results indicate that **1** can target both stem and bulk, differentiated rhabdomyosarcoma cells simultaneously, which represents a benefit not attainable by currently used antitumor chemotherapeutics.

## 4. Conclusions

In this work, some aspects of the anticancer effect of a new Ga(III) complex with cloxyquin ligands, namely tris(5-chloro-8-quinolinolato) gallium(III) (**1**), were investigated in cancer cells. Complex **1** shows potent antiproliferative activity in several human cancer cell lines, accompanied by significant selectivity to cancer over noncancerous cells. Notably, a prominent effect is found against highly aggressive and poorly treatable rhabdomyosarcoma cells, the most frequent soft tissue sarcomas of children. The data presented in this work show that **1** effectively penetrates the cell membrane and accumulates in the cell cytoplasm, likely in peroxisome-like structures or multivesicular bodies. Interestingly, **1** does not induce an apoptotic response in RD cells, but rather induces processes typical for autophagy and, most importantly, ferroptosis-like responses. The fact that the cell death caused by **1** in RD displays characteristic features of ferroptosis, as one of the possible mechanisms of antitumor effects of **1**, was proven from multiple aspects, indicating a unique cellular response to **1,** so far not described for any antitumor complex based on metal other than Fe. This may be related to the fact that gallium(III) shares several characteristics with iron(III) [13]. Gallium binds transferrin [47] and enters tumor cells via transferrin receptor 1, which results in disruption of iron metabolism [48]. The iron released from ferritin could accelerate the intracellular Fenton reaction and induce ferroptosis [49].

The molecular details of the mechanism by which Ga(III) complex with cloxyquin ligands perturbs intracellular iron homeostasis are not fully understood and may be the subject of further studies. However, it is known from the literature that gallium complexes comprehensively affect intracellular iron-dependent processes, for instance, inhibiting mitochondrial function and ribonucleotide reductase [9,48]. Additionally, gallium complexes can stimulate the increase of reactive oxygen species, upregulate hemoxygenase I, and affect signaling pathways in the cell. Current knowledge about the competing role of gallium complexes with iron has been exhaustively summarized in the review by Chitambar [50].

In conclusion, the results presented here give the evidence that **1** has a potential for further evaluation using *in vivo* models as chemotherapeutic agents for hardly treatable human rhabdomyosarcoma, particularly with respect to its very low toxicity in noncancerous cells, activity in both stem and differentiated cancer cells, and different modes of action compared to the metal-based antitumor drugs in clinical use or tests. As ferroptosis-driven therapy strategies are becoming increasingly promising in tumor treatment due to their brilliant tumor suppression [49, 51], the data presented here encourage the further evaluation of antitumor properties of **1**. In addition, this bolsters the effort to explore the biological relevance of Ga(III) coordination chemistry, providing access to novel Ga-based drugs and deepening the understanding of their anticancer activity.

## Figures and Tables

**Table 1:** IC_50_ values^a^ [*μ*M] for **1** in the various cell lines as determined by MTT assay after 72 h of incubation.

	MDA-MB-231	HCT116	518A2	RD	MCF-7	MRC5pd30
**1**	7 ± 3	3.2 ± 0.6	2.9 ± 0.2	1.3 ± 0.1	93 ± 12	149 ± 11
HClQ	12 ± 4	16 ± 2	7.3 ± 0.8	4.9 ± 0.3	7.8 ± 0.6	76 ± 6^b^
Ga(NO_3_)_3_	>200	>200	>200	> 200	>200	ND
Cisplatin	23 ± 3	5 ± 1	8.3 ± 0.6	2.0 ± 0.3	11 ± 3	12 ± 1
Carboplatin	198 ± 45	295 ± 7	55 ± 8	44.5 ± 0.8	81 ± 6	148 ± 13

^a^Data represent mean ± SD from at least three independent experiments, each performed in triplicate.

^b^Data taken from Ref. [22].

**Table 2:** Cellular internalization of Ga from **1** and Ga(NO_3_)_3_ in RD cells determined by ICP-MS.

Compound	**1**	Ga(NO_3_)_3_
ng Ga/10^6^ cells	14 ± 1	0.8 ± 0.3

Data represent a mean ± SEM from two independent experiments, each made in duplicate.

**Table 3:** IC_50_ values^a^ [*μ*M] determined in CD133-positive and CD 133-negative Rhabdospheres as determined by Cell TiterGlo 3D after 72 h incubation with indicated compounds.

	RD CD133+	RD CD133−
**1**	6 ± 2	8 ± 2
HClQ	13 ± 2	14 ± 4
Ga(NO_3_)_3_	>500	>500
Cyclophosphamide	>150^b^	41 ± 4^b^

^a^Data represent mean ± SD from three independent experiments, each performed in triplicate.

^b^Data taken from Ref. [26].

## Data Availability

All data used to support the findings of this study are included within the article.

## Supplementary Materials

Supplementary Materials Figure S1: Emission fluorescence spectra of **1** and free cloxyquin (HClQ). Figure S2: Flow cytometry-based profiling of autophagy in RD cells. Figure S3: Detection of ROS in RD cells by flow cytometry. Figure S4: Histograms of lipid peroxidation in RD cells analyzed by flow cytometry. Figure S5: Bright-field images of the rhabdospheres formed from RD CD133+ and RD CD133-cells.
