# Supplementary material for: Gallium(III) Complex with Cloxyquin Ligands Induces Ferroptosis in Cancer Cells and Is a Potent Agent against Both Differentiated and Tumorigenic Cancer Stem Rhabdomyosarcoma Cells
Source: Bioinorg Chem Appl. 2022 Apr 23;2022:3095749. doi: 10.1155/2022/3095749 (PMC9056256; doi:10.1155/2022/3095749)
Supplement: Supplementary Materials — Figure S1: Emission fluorescence spectra of 1 and free cloxyquin (HClQ). Figure S2: Flow cytometry-based profiling of autophagy in RD cells. Figure S3: Detection of ROS in RD cells by flow cytometry. Figure S4: Histograms of lipid peroxidation in RD cells analyzed by flow cytometry. Figure S5: Bright-field images of the rhabdospheres formed from RD CD133+ and RD CD133-cells. [file 3095749.f1.pdf]

Supporting Information

for

Gallium(III) complex with cloxyquin ligands induces ferroptosis in cancer cells and is a potent agent against both differentiated and tumorigenic cancer stem rhabdomyosarcoma cells

Monika Hreusova,[a] Vojtech Novohradsky,[a] Lenka Markova,[a] Hana Kostrhunova,[a] Ivan Potočňák,[b] Viktor Brabec[a,c] and Jana Kasparkova[a]*

[a]*Czech Academy of Sciences, Institute of Biophysics, Kralovopolska 135, 612 65 Brno, Czech Republic.*
[b]*Department of Inorganic Chemistry, Institute of Chemistry, P. J. Šafárik University in Košice, Moyzesova 11, 040 01 Košice, Slovakia*
[c]*Department of Biophysics, Faculty of Science, Palacký University, Šlechtitelů 27, 783 71 Olomouc, Czech Republic*

Table of Contents

Emission fluorescence spectra of **1** and free cloxyquin (HClQ) (Figure S1) S1

Flow cytometry-based profiling of autophagy in RD cells (Figure S2) S2

Detection of ROS in RD cells by flow cytometry (Figure S3) S3

Histograms of lipid peroxidation in RD cells analyzed by flow cytometry (Figure S4) S4

Bright-field images of the rhabdospheres formed from RD CD133+ and RD CD133- cells (Figure S5) S5

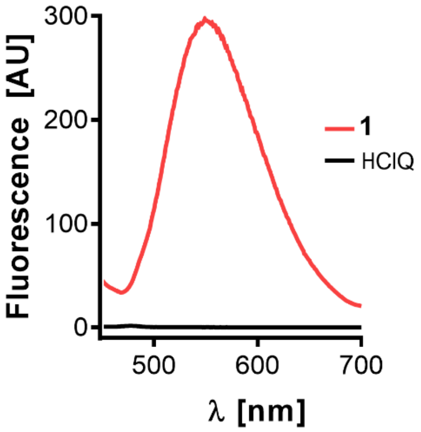

Figure S1: Emission fluorescence spectra of **1** and free cloxyquin (HClQ) at their 50 μM concentrations in Tris.Cl, pH 7.4 + 1 % DMSO; λ_{ex} = 410 nm.

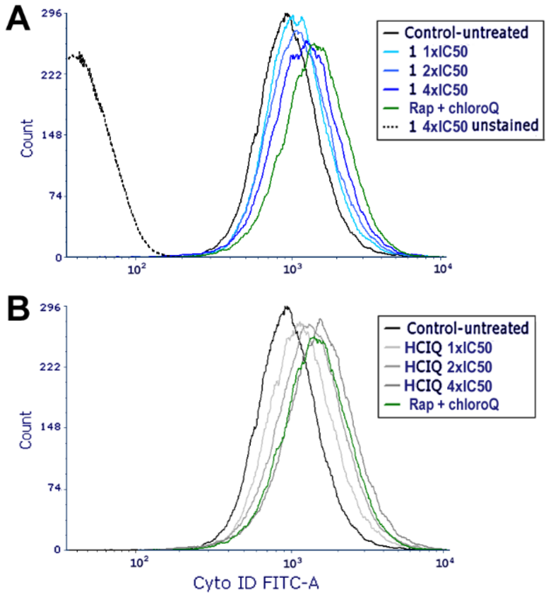

Figure S2: Flow cytometry-based profiling of autophagy in RD cells. Cells were untreated (black line) or treated with **1** (A) or cloxyquin (B) at their equitoxic concentrations corresponding to 1, 2, and 4- times the $IC_{50,72h}$ values. According to the manufacturer's recommendation, a mixture of Rapamycin (Rap, 1 µM) + Chloroquine (chloroQ, 100 µM) was included in the analyses as a positive control. After staining with CYTO-ID® Detection Reagent, cells were washed and analyzed by flow cytometry. Results are presented as histogram overlay, a representative histograms of two experiments are shown. Unstained controls were treated with **1** but not with CYTO-ID® reagent to verify that the resulting signal is not affected by the inherent fluorescence of **1**.

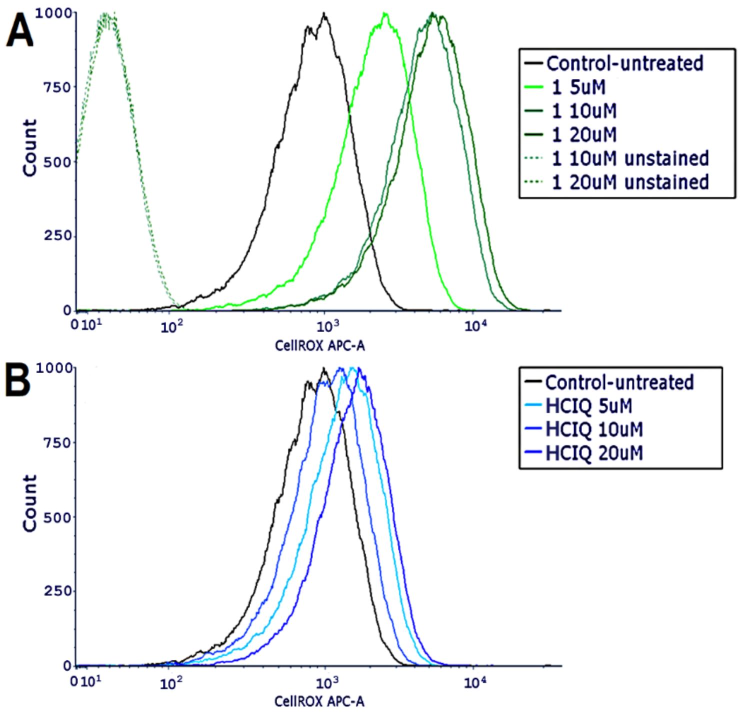

Figure S3: Detection of ROS in RD cells by flow cytometry. Cells were treated with increasing concentration of tested compounds for 3 h and subsequently stained with CellROX-deep red reagent for quantification of ROS. A) Cells treated with **1**. B) cells treated with CIQ ligand. Presented histograms are the representatives of two independent experiments. Unstained controls were treated with **1** but not with CellRox reagent to verify none fluorescent overlaps due to the inherent fluorescence of **1**.

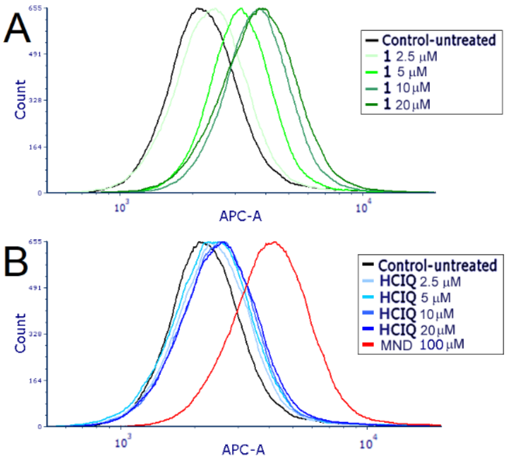

Figure S4: Representative histograms of lipid peroxidation in RD cells analyzed by flow cytometry. Cells were treated for 3 h with an increasing concentration of **1** (A) or HClQ (B). Positive control menadione (MND) was also included in the experiment (shown in panel B). Samples were stained with Bodipy^{TM} 665/676 lipid peroxidation sensor.

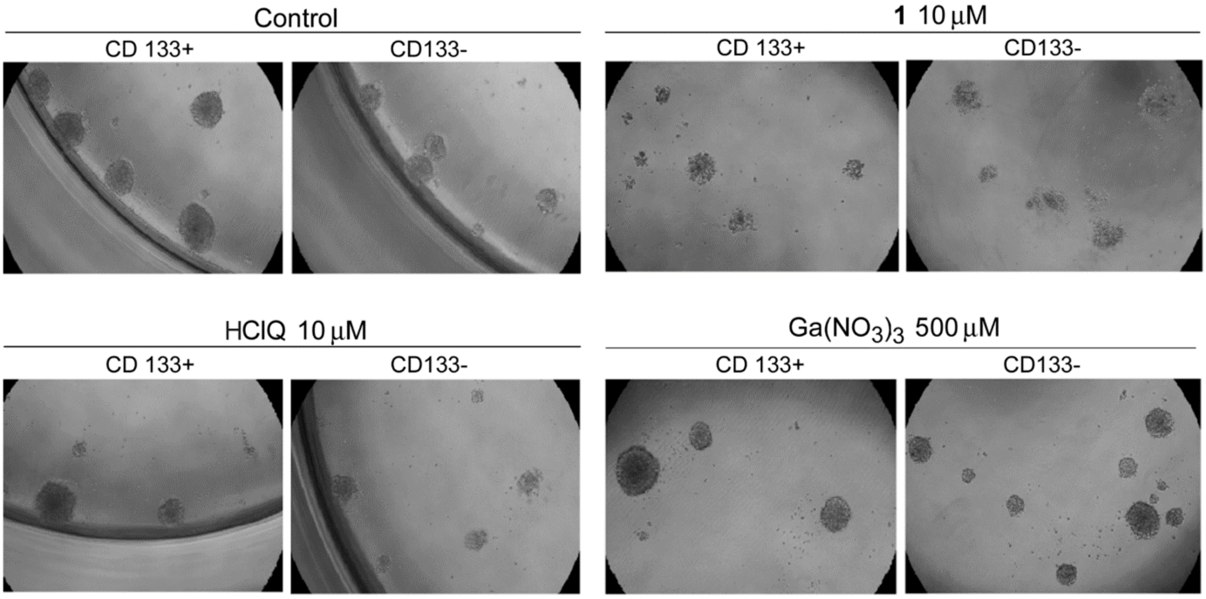

Figure S5: Representative bright-field images of the rhabdospheres formed from RD CD133+ and RD CD133- cells obtained after 72 h of the treatment with the indicated concentrations of **1** and ClQ.
